# Secondary below-knee amputation following open reduction and internal fixation of a closed pilon fracture: A case report and algorithm for management

**DOI:** 10.1097/MD.0000000000024791

**Published:** 2021-02-19

**Authors:** Daiqing Wei, Yangbo Xu, Feifan Xiang, Junwu Ye

**Affiliations:** a Department of Orthopaedics, The Affiliated Hospital of Southwest Medical University; b Sichuan Provincial Laboratory of Orthopaedic Engineering, Luzhou, Sichuan, China.

**Keywords:** amputation, case report, closed fracture, ischemia, pilon fracture

## Abstract

**Rationale:**

Despite significant advances in surgical techniques and implants, the clinical outcome of high-energy pilon fractures remains unsatisfactory, which continues to represent numerous challenges for orthopedic trauma surgeons.

**Patient concerns:**

A 62-year-old man injured his right ankle after falling from a 3 m high place. There were no open wounds or other complications.

**Diagnoses:**

According to the X-ray and CT scans, the patient was diagnosed with pilon fracture (type AO-43-C2) and lateral malleolus fracture of the right limb.

**Interventions:**

The patient was initially treated with calcaneal traction upon admission to a primary hospital. Five days after the injury, the patient underwent open reduction and internal fixation (ORIF) of the fracture and vacuum sealing drainage (VSD) for wound closure.

**Outcomes:**

The patient presented to our hospital on the 9th day after the first ORIF operation because of critical ischemia of the affected foot and distal lower leg. Blood circulation did not improve after a series of salvage treatments, and below-knee amputation was ultimately performed.

**Lessons:**

This is a rare case of complete ischemic necrosis following ORIF surgery of a closed pilon fracture due to iatrogenic damage. Standardized treatment that strictly follows the guidelines, instructions, or expert consensus should be promoted in this kind of complicated pilon fracture.

## 1 Introduction

Pilon fractures of the distal tibia involving the weight-bearing articular surface are common injuries, accounting for approximately 1% of all lower extremity fractures.^[1,2]^ It is usually caused by a high-energy axial force created from falling from a height or motor-vehicle-related traffic accident. There is a limited soft tissue envelope between the skin and bone at the ankle; therefore, injury to the surrounding soft tissue is often significant and the incidence of open fracture is relatively high. Despite significant advances in surgical techniques and implants, the clinical outcome of high-energy pilon fractures remains unsatisfactory, which is closely related to the anatomic reduction of the articular surface, correction of any mechanical malalignment, and the management of surrounding soft tissue injuries. It continues to represent numerous challenges for orthopedic trauma surgeons. This study presents a rare case of a closed pilon fracture that resulted in secondary below-knee amputation after ORIF surgery because of iatrogenic damage.

## 2 Case report

**Figure 1:**
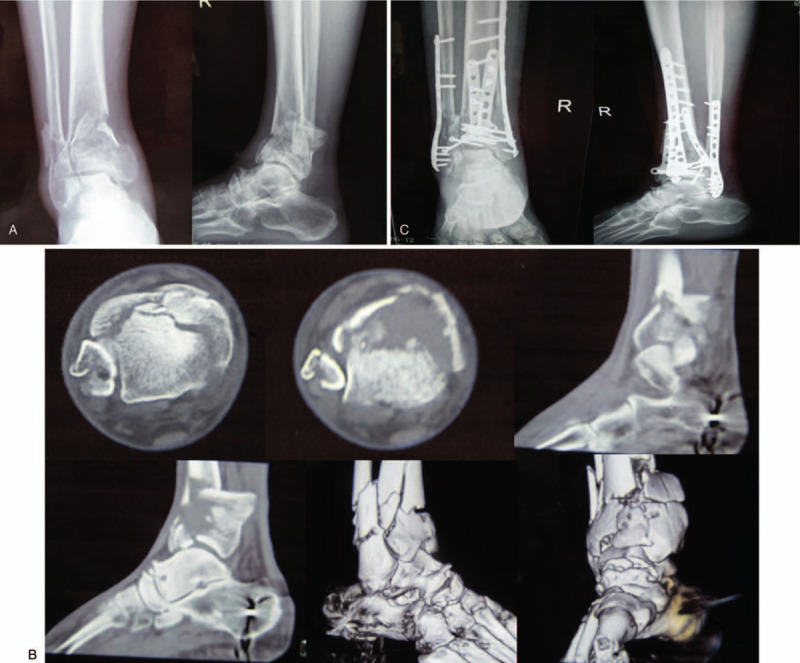
A. Preoperative X-rays. B. Preoperative CT scans and 3D reconstruction. C. Postoperative X-rays.

**Figure 2:**
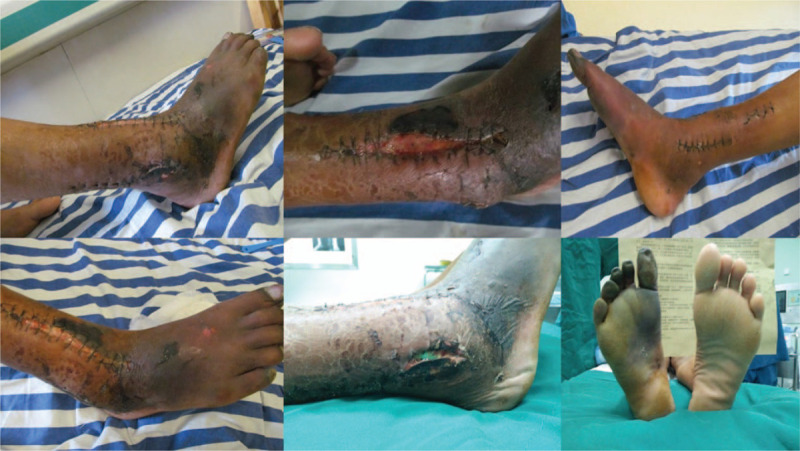
The affected foot and distal lower leg revealed critical ischemia at 9th day after ORIF surgery.

**Figure 3:**
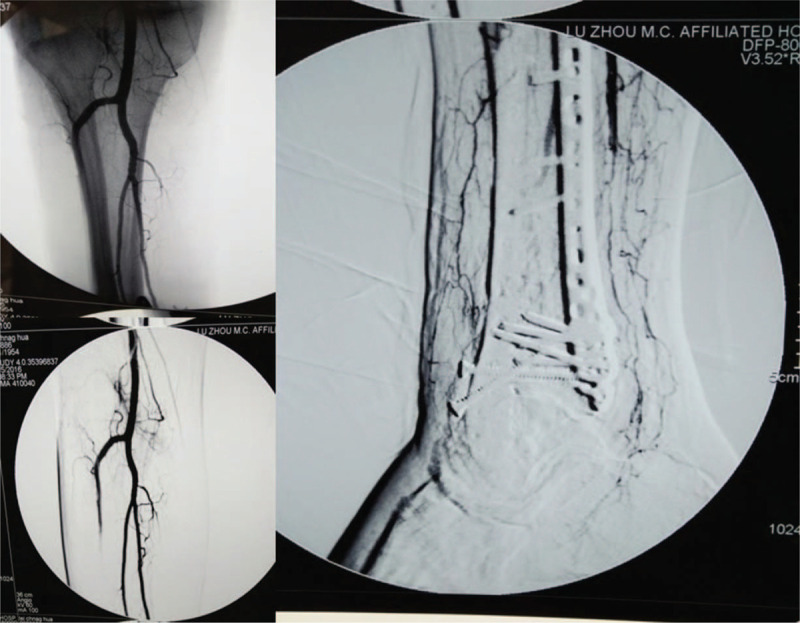
The DSA revealed vascular occlusion of upper third of the anterior tibial artery, occlusion of lower third of the posterior tibial artery and peroneal artery.

**Figure 4:**
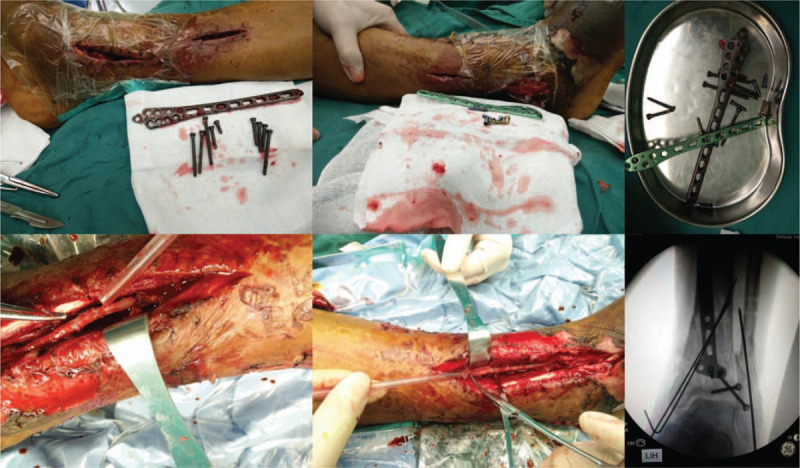
Exploration and decompression of ateries, and removing of the medial and lateral plates and screws.

**Figure 5:**
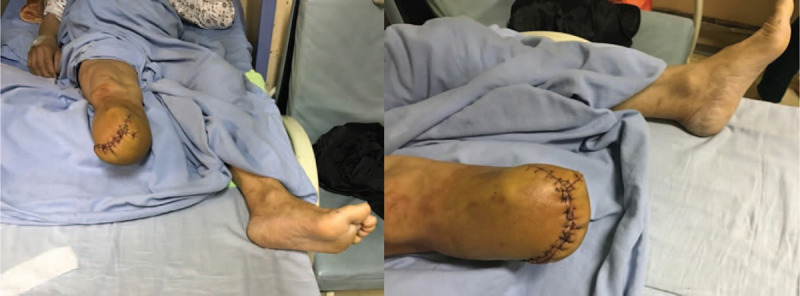
The patient was ultimately performed with below-knee amputation.

A 62-year-old male patient was admitted to a primary hospital because of right ankle injury after falling from a 3 m high place. The patient was diagnosed with pilon fracture (type AO-43-C2) and lateral malleolus fracture of the right limb according to the X-ray and CT scans (Fig. 1A, B), after which calcaneal traction was applied. Five days after injury, physical examination revealed mild swelling of the right ankle and no tension blisters. The anterior and posterior tibial arteries could be palpable without hemodynamic disorders and paresthesia. The patient underwent open reduction and internal fixation (ORIF) of the fracture under general anesthesia (Fig. 1C). It required approximately 5 hours for the surgical procedure because of the difficulty of reduction. The incision could not be closed completely after surgery; thus, vacuum sealing drainage (VSD), a type of negative pressure wound therapy, was used for wound closure. VSD was removed on the 6th day after surgery, and it was found that some skin edges of the anterolateral and posterolateral incisions were blackened and necrotic. The foot and toes had low skin temperature and dark skin color. Considering the poor blood supply to the right limbs, the patient underwent immediate debridement of the right ankle and immediate removal of the anterior plate and screws. Unfortunately, the condition has not changed for the better. Then the patient presented to our hospital for further treatment on the 9th day after the first ORIF operation. At the time of admission, the affected foot and distal lower leg revealed critical ischemia and nonpalpable palpation of the dorsalis pedis artery and posterior tibial artery (Fig. 2). Digital subtraction angiography (DSA) revealed vascular occlusion of the upper third of the anterior tibial artery, occlusion of the lower third of the posterior tibial and peroneal arteries, and a small amount of collateral circulation (Fig. 3). The patient was immediately treated with exploration and decompression of blood vessels and removal of the medial and lateral plates and screws, followed by thrombolytic and anticoagulant interventions to dissolve the clot. Surgical exploration demonstrated that the peroneal artery and anterior and posterior tibial arteries were continuous with long-segment embolisms (Fig. 4). Blood circulation did not improve after the operation, and below-knee amputation was ultimately performed due to persistent intense pain and extensive soft tissue necrosis of the threatened limb (Fig. 5). The procedures performed in this study were approved by the ethics committee of Affiliated Hospital of Southwest Medical University. Signed written informed consent was obtained from the patient.

## 3 Discussion

High-energy pilon fractures are associated with severe soft tissue injury, vascular injury, and bone defects, resulting in poor incision healing, infection, nonunion, traumatic arthritis, and other complications. Especially for some cases of Gustilo-Anderson type III C open fractures, limb salvage is still a big challenge for surgeons. Currently, the treatment technology for pilon fractures has improved, and the concept of biological fixation and soft tissue protection has been deeply rooted. The occurrence of ischemic necrosis after ORIF surgery is extremely rare, according to published data. Wynes et al reported a case of open pilon fracture resulting in delayed ischemia 19 days after the initial ORIF surgery, and transtibial amputation was ultimately performed.^[3]^ Bellezza et al reported a case of trimalleolar ankle fracture dislocation in an 86-year-old woman who underwent below-knee amputation after ORIF surgery.^[4]^ She had a type III A popliteal variant with an incidence of approximately 3.8%. Sandelin et al reported a 39-year-old woman who sustained a transtibial amputation after ORIF of a trimalleolar ankle fracture due to low-energy twisting injury.^[5]^ The vascular anomaly where a bilateral dominant peroneal artery as the only artery supplying vascularity to the foot was revealed in her.

In contrast to the above study, popliteal artery branching in this case is a usual I A pattern, which was confirmed by postoperative DSA. Palpable and symmetric pedal pulses were revealed on physical examination upon arrival at the initial hospital. The patient had no medical history of smoking, diabetes, peripheral vascular disease, or neuropathy. Unfortunately, the patient had a delayed vascular occlusion of the anterior tibial, posterior tibial, and peroneal arteries at different levels after ORIF surgery. It is not clear whether latent vascular injury was initiated after fracture or surgery. A few studies have illustrated that delayed infrapopliteal three-vessel occlusion may occur following lower leg closed fractures, even with a normal pulse examination.^[6,7]^ Blood vessel intimal lesions may only become detectable hours or days after the initial injury when some degree of thrombosis has occurred. There is limited evidence or clinical recommendations regarding closed pilon fractures and their association with vascular injury.

Excluding vascular factors, more importantly, the severity of this patient may be related to some iatrogenic factors. First, the operation time was inappropriate. The swelling of the affected limb peaked–3 to 5 days after injury, which is not suitable for complicated limb surgery in this period because of severe swelling of soft tissues. It is generally considered that for Tscherne grade 3 and AO type C pilon fractures, the contraindication period of ORIF is from 6 hours to 7 days after injury. During this period, the skin oxygen supply of the distal calf was very poor, and the incidence of soft tissue complications was extremely high.^[8]^ Second, the operation procedure is inappropriate. In this case, the preoperative soft tissue injury was not considered severe. A medial incision, anterolateral incision, and posterolateral incision were made in this case. However, it requires approximately 5 hours for the surgical procedure because of difficult reduction, which may be related to the selection of the approach and the operative skills. A long operation time and extensive separation of soft tissues may have a potential impact on blood vessels.^[9]^ The modified anteromedial incision combined with a posterolateral incision may be more appropriate for this case, according to our experience. The reduction from posterior, lateral to anterior with temporary Kirschner wires for fixation of small fragments and articular surface may be beneficial.^[10]^ At the very least, a successful staged protocol rather than a failed one-time operation is more advisable for this kind of complicated case. Third, there is an inappropriate postoperative management. Due to the swelling of soft tissues and the space effect of plates and screws, it was difficult to close the incisions, which may increase the risk of infection. Then, negative pressure wound therapy was adopted in the patient by using the VSD. No doubt that the VSD may play an important role in reducing the extent and complexity of the wound and promoting angiogenesis to the injured tissue. Nevertheless, palpation of the pedal pulses was not possible due to the coverage of the VSD, and this may result in the neglect of early symptoms of critical vascular problems. In this case, the patient was found to have insufficient blood supply immediately after the removal of VSD, causing an important delay in diagnosis and treatment.

**Figure 6:**
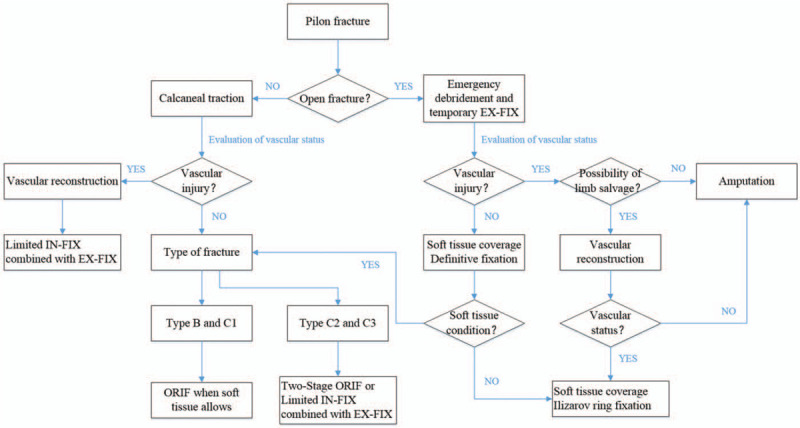
Algorithm for management of pilon fracture as used in the authors’ unit.

For this purpose, some important points should be carefully considered. First, the patient should be preoperatively evaluated. Comprehensive identification of the patient's medical condition should be performed before surgery, including the general condition, basic disease, and psychological status. Detailed knowledge of the fracture patterns is of prime importance because it is the foundation for making a precise surgical plan. Careful assessment of soft tissue injury is also extremely important in the clinical decision-making process for pilon fractures. Positive wrinkle signs and reepithelialization of blisters are indicators of decreased soft tissue swelling. Second, the vascular status should be preoperatively evaluated. Until now, there is no acceptable universal algorithm for the evaluation of vascular status in pilon fractures. The ankle brachial index, lower extremity Allen test, and color Doppler ultrasound are advised to routinely evaluate preoperative and postoperative vascular status. Color Doppler ultrasound is a cheap, convenient, and noninvasive method for checking blood flow in arteries and veins. Early identification of vascular injuries and early anticoagulant interventions may be beneficial. Third, optimal timing and method for surgery. Currently, there is no gold standard for the optimal timing and method for pilon fractures. It often depends on the patient's medical condition, fracture pattern, soft tissue condition, and the surgeon's experience. It is now widely accepted that for type B and C1 pilon fractures when soft tissue allows (often 7–14 days or within 24 hours after injury), ORIF surgery with or without a minimally invasive technique is desirable.^[11]^ For type C2 and C3 pilon fractures, a staged treatment protocol of initial ankle spanning external fixation with or without ORIF of the associated fibula, followed by definitive internal or external fixation at a later stage may have a better clinical outcome (Fig. 6).^[12]^ The success of a complicated pilon fracture surgery depends not only on the technical aspects of the operation, but also, to a larger extent, on the protection of the subsequent soft tissue and blood vessels.

This case exposed the insufficient education, training, and experience of some surgeons in primary hospitals. It warns against attempts to perform surgery based only on assumptions. Standardized treatment that strictly follows the guidelines, instructions, or expert consensus should be promoted in this kind of complicated pilon fracture.

## Author contributions

**Conceptualization:** Junwu Ye.

**Project administration:** Feifan Xiang.

**Writing – original draft:** Daiqing Wei, Yangbo Xu.

**Writing – review & editing:** Daiqing Wei, Junwu Ye.

## References

[1] Mauffrey C Vasario G Battiston B. Tibial pilon fractures: a review of incidence, diagnosis, treatment, and complications. Acta Orthop Belg 2011;77:432–40. 21954749

[2] Zelle BA Dang KH Ornell SS. High-energy tibial pilon fractures: an instructional review. Int Orthop 2019;43:1939–50. 31093715 10.1007/s00264-019-04344-8

[3] Wynes J Kirksey L. Assessing vascular status and risk of latent ischemia with ankle fracture: a case report and algorithm for treatment. J Foot Ankle Surg 2014;53:353–5. 24589135 10.1053/j.jfas.2014.01.001

[4] Bellezza PA Elliott E Conlee T. Aplastic posterior tibial artery in the presence of trimalleolar ankle fracture dislocation resulting in below-the-knee amputation. J Foot Ankle Surg 2017;56:92–7. 27839661 10.1053/j.jfas.2016.08.006

[5] Sandelin H Tukiainen E Ovaska M. Amputation following internal fixation of an ankle fracture via the posterolateral approach - a case report. Acta Orthop 2017;88:358–60. 27892754 10.1080/17453674.2016.1262679 PMC5434610

[6] Causey MW Oguntoye MO Miller S. Limb salvage after delayed diagnosis for blunt traumatic infrapopliteal occlusion. J Vasc Surg 2010;52:734–7. 20570464 10.1016/j.jvs.2010.03.065

[7] Evangelista PJ Evangelista LM Evangelista GT. Delayed complete limb ischemia following a closed tibial shaft fracture. Am J Orthop 2013;42:569–72. 24471148

[8] Bear J Rollick N Helfet D. Evolution in management of tibial pilon fractures. Curr Rev Musculoskelet Med 2018;11:537–45. 30343399 10.1007/s12178-018-9519-7 PMC6220009

[9] Kottmeier SA Madison RD Divaris N. Pilon fracture: preventing complications. J Am Acad Orthop Surg 2018;26:640–51. 30134307 10.5435/JAAOS-D-17-00160

[10] Jacob N Amin A Giotakis N. Management of high-energy tibial pilon fractures. Strategies Trauma Limb Reconstr 2015;10:137–47. 26407690 10.1007/s11751-015-0231-5 PMC4666229

[11] Saad BN Yingling JM Liporace FA. Pilon fractures: challenges and solutions. Orthop Res Rev 2019;11:149–57. 31576179 10.2147/ORR.S170956 PMC6765393

[12] Tomás-Hernández J. High-energy pilon fractures management: state of the art. EFORT Open Rev 2017;1:354–61. 28461913 10.1302/2058-5241.1.000016 PMC5367607

